# A Case of Angiomyolipoma Rarely Located in the Larynx

**DOI:** 10.1155/2011/427074

**Published:** 2011-10-09

**Authors:** Hulya Eyigor, Dinc Suren, Ustun Osma, Cem Sezer, Mustafa Deniz Yilmaz

**Affiliations:** ^1^Department of ENT Head and Neck Surgery, Antalya Education and Research Hospital, 07100 Antalya, Turkey; ^2^Department of Pathology, Antalya Education and Research Hospital, 07100 Antalya, Turkey

## Abstract

Angiomyolipoma is a rare benign mesenchymal tumor, which is mostly renal in origin. A sixty-year-old male patient with the diagnosis of angiomyolipoma located in the larynx has been presented here, and the literature is reviewed.

## 1. Introduction

Angiomyolipoma is a rare benign mesenchymal tumor which is most frequently located in the kidney. Accompanying tuberous sclerosis syndrome is present in 50% of angiomyolipoma patients. Angiomyolipoma has been reported in 80% of tuberous sclerosis patients [1]. The second most common location of the tumor is the liver, followed by the abdomen, genital organs, the heart, the mediastinum, lung, skin, head, and the neck [1–4]. In the head and neck region, angiomyolipoma has frequently been reported to be located in the oral cavity, nasal cavity, and nasopharynx [2, 4, 5]. Our case is an angiomyolipoma that had originated from the vocal process of the arytenoid and posterior of the the vocal cord. Only a few laryngeal angiomyolipoma cases are available in the literature [6–8].

## 2. Case Report

**Figure 1:**
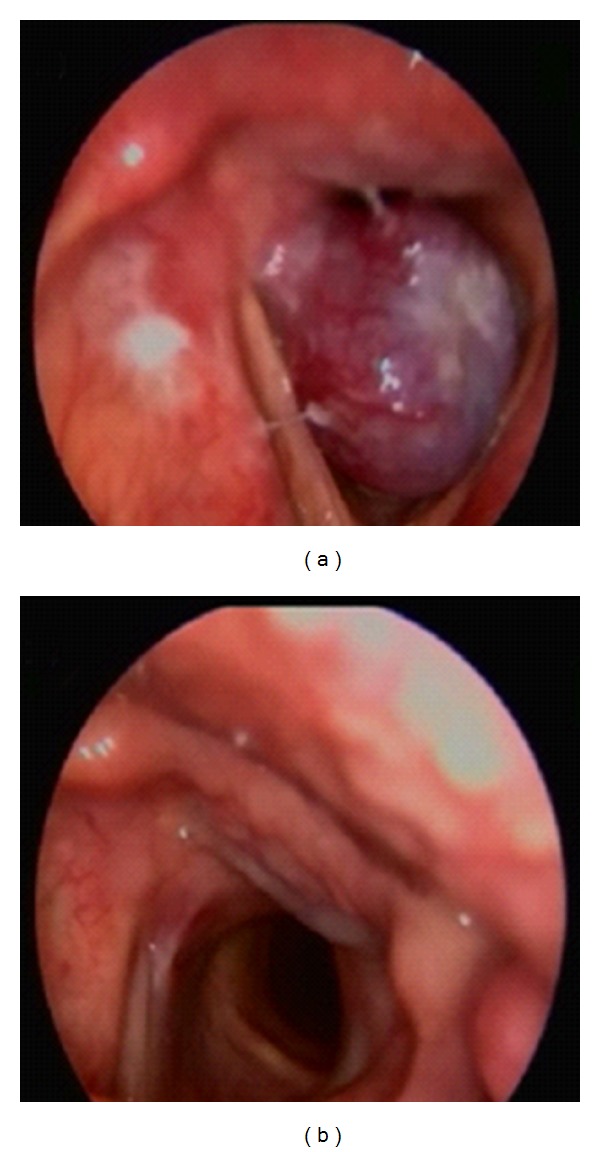
(a) Preoperative Videolarygoscopy. A smooth submucosal tumor located in the left arytenoid. (b) Videolaryngoscopic appearance at the 4 postoperative week.

A 60-year-old male patient presented to our clinic with the complaints of hoarseness persisting for one year. He had respiratory distress appearing in the last 3-4 months. His medical history was unremarkable except for history of smoking (1 package per day for 40 years). On his videolaryngoscopic examination, a 1.5 cm vascularized, pedunculated polypoid lesion, originating from the posterior half of the vocal cord and vocal process of the arytenoid, was observed. The mass was obstructing the endolarynx, subtotally (Figure 1(a)). A tracheotomy was performed under general anesthesia to secure the airway. The mass was totally excised by endolaryngeal microsurgery. Postoperative videolaryngoscopic examination on the 4th month revealed a healed incision with no residual or recurrent mass (Figure 1(b)).

## 3. Histopathological Findings

**Figure 2:**
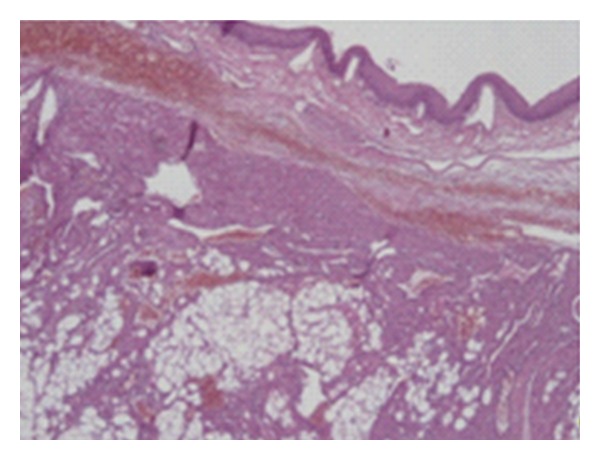
The tumour is composed of adipose tissues, smooth muscles, and vessels (Hematoxylin and eosin; magnification ×40).

**Figure 3:**
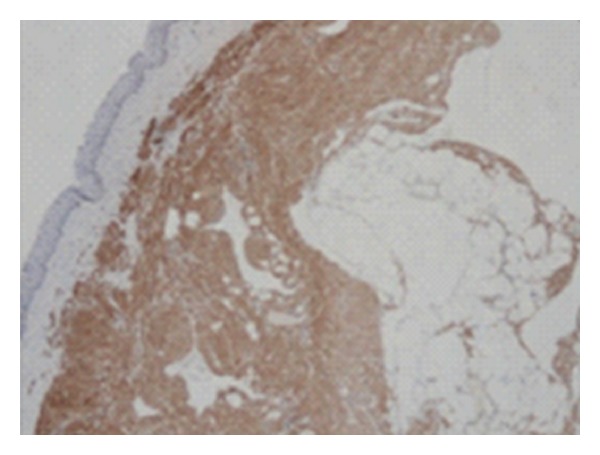
Smooth muscle component showing immunoreactivity to SMA (SMA; magnification ×40).

The surgical specimen was fixed in a 10% neutral buffered formaldehyde solution and embedded in paraffin. On routine hematoxylin and eosin stains, microscopic examination revealed a tumour composed of an intimate admixture of tortuous blood vessels, bundles of smooth muscle, and mature adipose tissue (Figure 2). Smooth muscle actin (SMA) (Figure 3) and desmin immunostaining were detected in the smooth muscle component. CD34, an endothelial marker, stained the vessel endothelium. Tumor cells were devoid of melanocytic markers such as HMB-45 and Melan-A, which are positive in renal angiomyolipoma. The pathological findings were concordant with angiomyolipoma.

## 4. Discussion

Angiomyolipoma is a rare benign mesenchymal tumor composed of varying proportions of mature lipoid tissue, smooth muscle fibers, and vessels with thick walls. It is most commonly seen in women. The most common form of angiomyolipoma is renal angiomyolipoma. The lesion, which is asymptomatic and usually detected incidentally, is also seen in systemic diseases like tuberous sclerosis [2]. While 50% of renal angiomyolipoma cases are seen together with tuberous sclerosis syndrome, extrarenal cases are sporadic [9]. Mental retardation, epilepsy, and cutaneous lesions such as adenoma sebaceum are present in tuberous sclerosis syndrome. There were no additional findings suggesting tuberous sclerosis in our case, similar to the other laryngeal angiomyolipoma cases in the literature.

The most commonly known mesenchymal tumors of the larynx are lipomas, chondromas, vascular tumors, and paragangliomas. Schwannoma originates from the superior laryngeal nerve and may usually be encountered in a pedunculated form in the aryepiglottic plica or submucously. Chondromas may usually be located in the posterior lamina of the cricoid cartilage and cause a subglottic tumefaction [10, 11]. In the two previous laryngeal angiomyolipoma cases in the literature, the lesion was reported to have originated from the aryepiglottic fold, partially obstructing the vocal cords [6, 7]. In our case, a 1.5 cm size, vascularized, pedunculated polypoid lesion with a smooth surface originating from the vocal process of the arytenoid and posterior half of the vocal cord and subtotally obstructing the endolarynx was observed.

While laryngeal angiomyolipoma may be asymptomatic, symptoms may vary according to the size and location of the lesion [7]. The most common symptoms in previous cases have been snoring, dyspnea, dysphonia, dysphagia, and odynophagia [6, 7]. The primary symptoms in our case were dysphonia and dyspnea causing the patient to refer to a physician. The approach to benign laryngeal masses varies depending on the location and size of the lesion. While small lesions may be excised through endolaryngeal microsurgery, an external approach (laryngofissure, lateral pharyngotomy, or thyrotomy) may be used for large lesions [12]. While both methods have been used in previous cases, in our patient, the lesion was totally excised by the endolaryngeal microsurgery method.

Endoscopic preoperative histopathological diagnosis is difficult due to submucosal development of the tumor. Other tumors of adipose tissue (angiolipoma, liposarcoma) and angioleiomyoma should initially be taken into consideration in the microscopic differential diagnosis. Angiolipoma lacks myoid differentiation. Lipoid component does not exist in an angioleiomyoma. In order not to misdiagnose a liposarcoma, lipoid component must be searched carefully for lipoblasts. It has been reported that HMB-45 positivity in angiomyolipoma when located in the kidneys is related to tuberous sclerosis. In our case, the tumor did not take up the HMB-45 dye, as well as another melanocytic marker Melan-A [3–5]. 

Melanocytic marker positivity is constitutional for angiomyolipomas. It was not observed in our case as well as the other reported cases. However, the presence of adipose and muscle tissue and tortoise vessels allows us to make the diagnosis of angiomyolipoma easily [6]. HMB-45 positivity was neither observed in our case nor in other cases of nasal angiolipomas in the literature [3]. In light of this finding, we can speculate that nasal angiomyolipomas have different immunohistochemical properties than their counterparts.

In conclusion, we aimed to emphasize the importance of the histopathological diagnosis for differentiation from other laryngeal pathologies by presenting a laryngeal angiomyolipoma case rarely encountered in the literature.
